# COVID-19 and Hypertension: The What, the Why, and the How

**DOI:** 10.3389/fphys.2021.665064

**Published:** 2021-05-03

**Authors:** Shah-Abas Muhamad, Azizah Ugusman, Jaya Kumar, Dominik Skiba, Adila A. Hamid, Amilia Aminuddin

**Affiliations:** ^1^Department of Physiology, Faculty of Medicine, Universiti Kebangsaan Malaysia Medical Centre, Kuala Lumpur, Malaysia; ^2^Institute of Genetics and Animal Biotechnology, Polish Academy of Sciences, Magdalenka, Poland

**Keywords:** coronavirus, COVID-19, SARS-CoV-2, hypertension, endothelial dysfunction, renin angiotensin system, angiotensin converting enzyme 2

## Abstract

It has been a year since the coronavirus disease 2019 (COVID-19) was declared pandemic and wreak havoc worldwide. Despite meticulous research has been done in this period, there are still much to be learn from this novel coronavirus. Globally, observational studies have seen that majority of the patients with COVID-19 have preexisting hypertension. This raises the question about the possible relationship between COVID-19 and hypertension. This review summarizes the current understanding of the link between hypertension and COVID-19 and its underlying mechanisms.

## Introduction

In December 2019, a novel coronavirus emerged from the Huanan seafood market in Wuhan City of Hubei Province of China, known to trade exotic live animals and their parts. The chronology of the outbreak started with certain market-goers developed severe infection of the lower respiratory tract in the form of pneumonia of unknown etiology (Rothan and Byrareddy, 2020). Provisionally the disease was called coronavirus disease 2019 (COVID-19). On January 7, 2020, the Chinese authorities found that the organism was a new strain of the severe acute respiratory syndrome coronavirus 1 (SARS-CoV-1), which was the virus responsible for the SARS pandemic in 2003 (World Health Organization, 2020). The COVID-19 virus was then renamed SARS-CoV-2. Henceforward, the virus has rapidly spread across continents and by March 11, 2020 the World Health Organization (WHO) declared it a pandemic.

With an exponential rise of COVID-19 cases worldwide, observational studies identified that patients with preexisting co-morbidities such as hypertension, diabetes mellitus and cardiovascular diseases are more susceptible to COVID-19 infection and its complications (Kunal et al., 2020). This statement is further supported by a meta-analysis involving seven studies with 1,576 participants, which shows that the most prevalent comorbidities are hypertension and diabetes, followed by cardiovascular disease and respiratory disease (Yang et al., 2020).

## Severe Acute Respiratory Syndrome Coronavirus-2

The name coronavirus is derived from the Latin word “corona” meaning crown, denoting the large spike protein (S protein) molecules on the virion’s surface that create a crown-like shape (González et al., 2003). Most of the pathogenic human coronaviruses are associated with mild clinical symptoms (Su et al., 2016), with two notable exceptions, SARS-CoV or SARS-CoV-1, and Middle East respiratory syndrome coronavirus (MERS-CoV). SARS-CoV-1 is a novel beta coronavirus that emerged in Guangdong, southern China in November 2002 (Peiris et al., 2004), which caused more than 8,000 human infections and 884 deaths in 37 countries from 2002 to 2003 (Chan-Yeung and Xu, 2003). Meanwhile, MERS-CoV was first detected in Saudi Arabia in 2012 (Zaki et al., 2012) and was responsible for 2,494 laboratory-confirmed cases of infection, including 858 fatalities since September 2012, and 38 deaths following a single introduction into South Korea (Lee et al., 2017).

According to genome sequencing, SARS-CoV-2 is approximately 82% identical to human SARS-CoV-1 and approximately 50% identical to MERS-CoV (Lu et al., 2020). Several phylogenetic analyses suggested that bats are the most probable animal reservoir for SARS-CoV-2. Both SARS-CoV-1 and MERS-CoV are transmitted from bats to other intermediate hosts such as palm civets, pangolins, or dromedary camels before finally transmitted to humans. However, the intermediate host for SARS-CoV-2 is still uncertain (Xie and Chen, 2020). Interestingly, household pets such as cats and dogs have also been reported to be infected, however, there is no evidence for SARS-Cov-2 transmission from them to human. In contrast, other domestic animals such as pigs and poultry are unsusceptible to SARS-CoV-2 (Kiros et al., 2020). However, other farm animals such as minks have recently been found to be infected with SARS-CoV-2. Moreover, the viral sequence similarity of infected minks and farm employees suggested a mink origin of SARS-Cov-2 infection in human (Oreshkova et al., 2020). In regions with affected mink farms, the number of COVID-19 infections had increased and 17 million of the animals were intended to be culled to prevent the spread of the virus (Mallapaty, 2020).

## Renin-Angiotensin System

**FIGURE 1:**
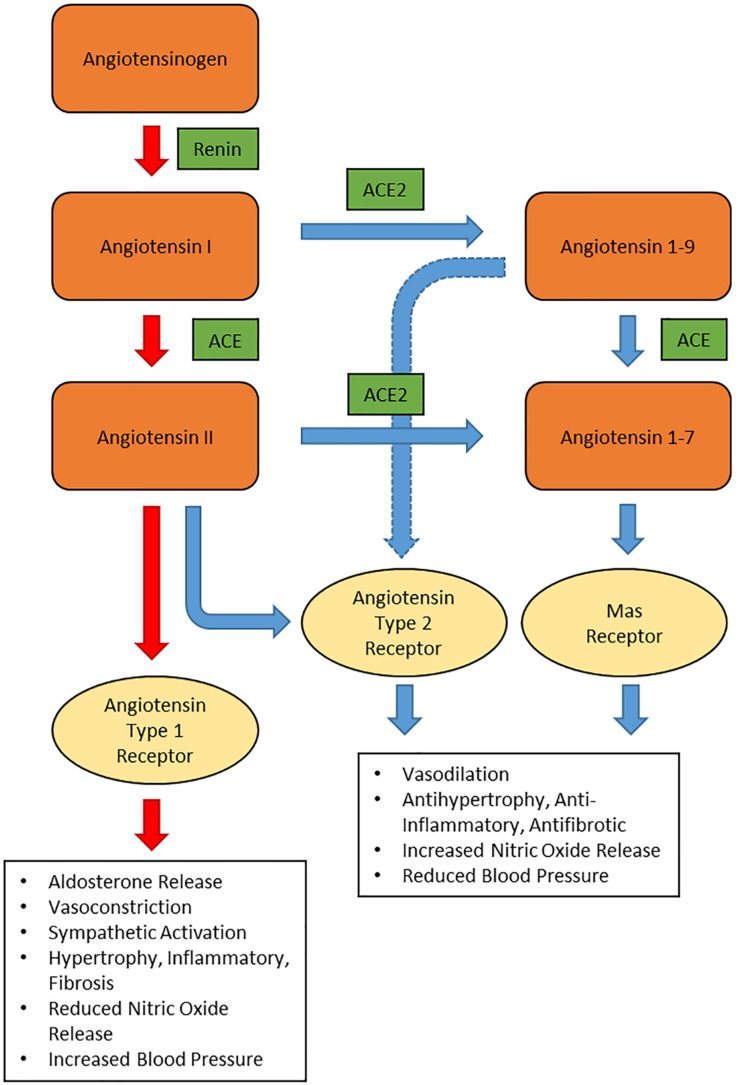
Schematic representation of renin-angiotensin system. ACE: angiotensin converting enzyme, ACE2: angiotensin converting enzyme 2.

To understand the pathogenicity of SARS-CoV-2 in hypertensive patients, one must first look into the renin-angiotensin system (RAS) function and expression of angiotensin converting enzyme 2 (ACE2). The RAS (Figure 1) is an important hormonal mechanism governing the hemodynamic stability by regulating the blood pressure, fluid volume, and sodium-potassium balance. Renin is synthesized by the juxtaglomerular cells in the kidneys and released into the circulation. Renin then catalyzes the cleavage of the glycoprotein angiotensinogen, generating angiotensin 1 (Ang I). Ang I is then cleaved by ACE to form angiotensin II (Ang II); the main effector in the RAS. Ang II binds to angiotensin II type 1 receptor (AT_1_R), triggering the synthesis and secretion of aldosterone in the adrenal cortex. Through specific actions on the distal nephron of the kidney, aldosterone promotes sodium reabsorption and water retention, ultimately increasing the blood pressure (Muñoz-Durango et al., 2016). In the vasculature, Ang II acts on the AT_1_R, resulting in diminished nitric oxide and vasoconstriction (Sparks et al., 2014). Ang II via AT_1_R also promotes other effects such as inflammation, fibrosis and production of reactive oxygen species (ROS). This process is referred as the RAS conventional axis, whereby Ang II exhibits its harmful effects through AT_1_R (Mori et al., 2020).

In the non-conventional axis of RAS, ACE2 converts Ang I to Angiotensin 1-9 (Ang 1-9) and Ang II to angiotensin 1-7 (Ang 1-7). Ang 1-7 binds to Mas receptor (Mas) and triggers the activation of endothelial nitric oxide synthase (eNOS) to release nitric oxide, a potent vasodilator (Mordwinkin et al., 2012). Apart from releasing nitric oxide, binding of Ang 1-7 to Mas also provides several protective effects such as such as anti-fibrosis, anti-vascular smooth muscle cell proliferation, and anti-inflammation (Ingelfinger, 2009; Santos et al., 2017; Skiba et al., 2017). Meanwhile, Ang 1-9 is a vasoactive peptide that has a protective effect on the heart and blood vessels in hypertensive patients (Ocaranza et al., 2014). In experimental model of hypertension, Ang 1-9 reduces the blood pressure and oxidative stress in the heart and aorta of hypertensive rats. Moreover, Ang 1-9 has a vasorelaxant effect and it reduces cardiac fibrosis and hypertrophy. These effects are mediated by angiotensin II type 2 receptors (AT_2_R) but not Mas (Ocaranza et al., 2014). Meanwhile, binding of Ang II to AT_2_R produces similar vasodilatory effects as binding of Ang 1-7 to Mas and counteracts the vasoconstriction produced by binding to AT_1_R (Ocaranza and Jalil, 2012). However, in adult tissues, the concentration of AT_2_R is less abundant and hardly detected in many cellular systems, including those of cardiovascular relevance (Steckelings et al., 2005).

## Role of ACE2 in SARS-CoV-2 Infection

ACE2 is a mono-carboxypeptidase with a single enzymatic binding site that acts as a key counter-regulatory component of the conventional RAS (South et al., 2019). ACE2 is an important counter-regulatory pathway within the RAS that is initiated through the cleaving of vasoconstrictive Ang II into the vasodilator Ang 1-7. ACE2 is expressed on the surface of endothelial and epithelial cells in membrane-bound and soluble forms throughout the body including the kidneys, heart, gastrointestinal tract, and especially abundant in the lungs (Donoghue et al., 2000; Hamming et al., 2004). Additionally, ACE2 is largely expressed in the nasal and oropharyngeal epithelium, where the SARS-CoV-2 entrance occurs (Lanza et al., 2020).

Angiotensin converting enzyme 2 is a type I transmembrane protein which consists of 805 amino acids with an extracellular N-terminal domain and an intracellular C-terminal tail. The N-terminal domain has a zinc-binding motif (HEMGH domain) which is essential for both formation of Ang 1-7 and SARS-CoV-2 Spike protein (S-protein) binding. The S-protein of coronavirus is responsible for binding to host receptors. The S-protein forms a trimer on the virus surface with each monomer harboring a receptor binding domain (RBD) that interacts with a particular receptor on the host cell (Li, 2016). The RBD of the SARS-CoV-2 S-protein supported a strong interaction with human ACE2 molecules (Xu et al., 2020). *In vitro* study showed that HeLa cells expressing ACE2 were more susceptible to SARS-CoV-2 infection compared to cells without ACE2 (Wan et al., 2020). Thus, the findings suggested that ACE2 is the functional receptor for cellular entry of SARS-CoV-2.

**FIGURE 2:**
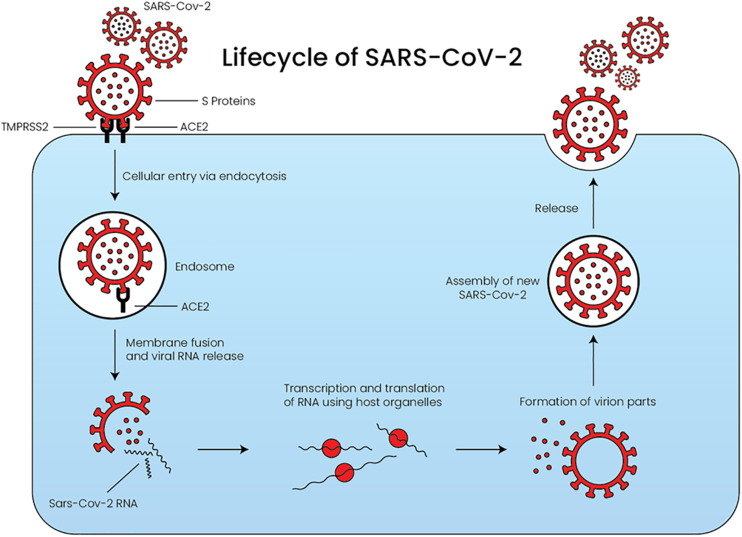
The lifecycle of SARS-CoV-2 starting from the penetration of the virus into the cell until its release. The virus requires both ACE2 and TMPRSS2 to facilitate its entry. ACE: angiotensin converting enzyme, TMPRSS2: transmembrane protease serine 2.

The life cycle of SARS-CoV-2 consists of five major steps, as depicted in Figure 2 (Yuki et al., 2020). During the initial attachment phase, the infection begins when the SARS-CoV-2 S-protein binds to ACE2. This is followed by the penetration phase, in which both the virus and ACE2 enter the cell via endocytosis or membrane fusion after cleavage of ACE2 by transmembrane protease serine 2 (TMPRSS2). TMPRSS2 is an essential protease required by SARS-CoV-2 to facilitate its entry (Amraei and Rahimi, 2020). Recently, TMPRSS2 and ACE2 co-expression was reported among a subset of type II pneumocytes, which explains why SARS-CoV-2 infection highly affects the lung function (Ziegler et al., 2020). During the biosynthesis phase, the SARS-CoV-2 structure and genome are synthesized using the host cellular organelles’ machinery. Subsequently, during the maturation phase, the viral structures are assembled into new SARS-CoV-2 in the cells exponentially. Finally, the newly synthesized SARS-CoV-2 are released into the circulation by exocytosis, and the cycle will be repeated (Yuki et al., 2020).

Following viral endocytosis, ADAM metallopeptidase domain 17 (ADAM17) activity increases which results in the shedding of the ectodomain of ACE2 from the cell surface (Hoffmann et al., 2020). ACE2 removal following SARS-CoV-2 infection may lead to a physiological imbalance between ACE and ACE2 activity that favors ACE, hence leading to worsening of the disease. ACE2 shedding and internalization results in increased Ang II activity, as less ACE2 are available to cleave Ang II into Ang 1-7. Ultimately, this leads to a shift from the ACE2/Ang 1-7/Mas axis to the ACE/Ang II/AT_1_R axis (Amraei and Rahimi, 2020). Pulmonary vasoconstriction and raised blood pressure lead to pulmonary edema and eventually the endpoint complication; acute respiratory distress syndrome (ARDS), and death (Kuba et al., 2005; Klhůfek, 2020).

In ARDS, it has been demonstrated that pulmonary expression of ACE2 was decreased whereas ACE was elevated (Wösten-van Asperen et al., 2013). This leads to a shift from the ACE2/Ang 1-7/Mas axis to the ACE/Ang II/AT_1_R axis with cardiovascular consequences. Therefore, it is hypothesized that the administration of Ang 1-7 to the infected organism may protect from the severe outcome of SARS-CoV-2 infection, especially in hypertensive patients (Magalhaes et al., 2020). Recently, a few clinical trials related to the administration of Ang 1-7 to COVID-19 patients are registered at www.clinicaltrials.gov (NCT04332666, NCT04375124, NCT04570501, NCT04605887, and NCT04633772) to further investigate this hypothesis.

Even though ACE2 has been recognized as the receptor for SARS-CoV-2, there might be other receptors or co-receptors for this virus that are yet to be discovered. For instance, ACE2 knockout mice had a reduced incidence of SARS-CoV infection but the absence of ACE2 did not completely prevent the infection from occurring (Kuba et al., 2005). This suggested that there could be other receptors involved in a viral invasion. Intracellular pathogens usually attach to more than one host cell surface structure that functions as the viral receptor. Carbohydrates, proteins, integrins, and membrane-bound ACE2 are common receptors used by viruses (Maginnis, 2018).

Recently, CD147, a transmembrane glycoprotein that belongs to the immunoglobulin superfamily, was identified as a novel receptor for SARS-CoV-2 (Wang et al., 2020b). CD147 is abundantly expressed in the epithelium and immune cells and plays a role in inflammatory processes and virus host cell entry (Radzikowska et al., 2020). Coincidentally, CD147 was involved in SARS-CoV infection, and CD147 antagonistic peptides have an inhibitory effect on SARS-CoV (Chen et al., 2005). Another possible receptor is CD209L (L-SIGN), which is a type II transmembrane glycoprotein identified as the receptor of SARS-CoV (Jeffers et al., 2004). Considering that SARS-CoV-2 has a similarity to SARS-CoV, CD209L is another potential receptor for SARS-CoV-2. In short, besides ACE2, there are several other potential receptors for SARS-CoV-2.

## Hypertension as a Risk Factor for Severe COVID-19 Outcome

Hypertension has gained popularity among researchers owing to its over-representation among COVID-19 patients (Schiffrin et al., 2020). The observational and retrospective studies conducted near Wuhan area have reported that hypertension is the most common co-morbidity observed in patients affected by COVID-19, ranging from 15 to 30% (Wang et al., 2020a; Zhang et al., 2020c; Zhou et al., 2020). In one of the largest studies conducted between December 11, 2019 and January 29, 2020 in Wuhan with data encompassing on 1,099 COVID-19 patients, 165 patients (∼15%) had high blood pressure. The same study also reported a total of 23.7% of hypertensive patients had higher disease severity compared to 13.4% of normotensive subjects. Whereas, 35.8% of hypertensive patients reached the composite endpoint of intensive care unit (ICU) admission, mechanical ventilation, and death compared to just 13.7% of normotensive patients (Guan et al., 2020b).

A separate study in China investigating 138 laboratory-confirmed COVID-19 patients reported similar high prevalence (31.2%) of hypertension among the patients. The researchers also found that 58.3% of hypertensive patients with COVID-19 infection were admitted to ICU compared to 21.6% of patients with normal blood pressure (Wang et al., 2020a). In a cohort of 1,590 patients from 575 hospitals, preexisting hypertension was independently associated with severe COVID-19 (hazard ratio 1.575, 95% CI: 1.07–2.32) (Guan et al., 2020a). Altogether, the findings indicate that hypertensive patients have a higher risk of developing severe outcome from COVID-19.

The mechanisms that link preexisting hypertension and COVID-19 are yet to be fully elucidated but it could be related to endothelial dysfunction and RAS imbalance. The conventional RAS (ACE/Ang II/AT_1_R) axis activation in parallel with non-conventional (ACE2/Ang 1-7/Mas) axis down-regulation was proposed to be the underlying factors leading to severe COVID-19 outcome in hypertension (Lanza et al., 2020; Vieira et al., 2021). Besides, hypertension is associated with endothelial dysfunction and a pro-inflammatory state, which includes higher levels of Ang II, chemokines, and cytokines, including interleukin-6 (IL-6) and tumor necrosis factor-α (TNF-α) (de Miguel et al., 2015). Therefore, RAS imbalance that favors the pro-inflammatory state is proposed to be the center of COVID-19 pathophysiological mechanisms (Costa et al., 2020).

## Endothelial Dysfunction as a Link Between Hypertension and SARS-CoV-2 Infection

The term endothelial dysfunction is used to describe the reduced bioavailability of nitric oxide or imbalance between the endothelium-derived relaxing and constrictor factors (Konukoglu and Uzun, 2017). In other words, endothelial dysfunction happens when the vascular walls become stiffer and have diminished vasorelaxation properties (Rogier van der Velde et al., 2015). Endothelial dysfunction is the common denominator for most COVID-19 co-morbidities such as hypertension, diabetes, and obesity. The presence of endothelial dysfunction in hypertensive patients was first demonstrated in the early 1990s through findings such as impaired endothelium-dependent vasodilation of the forearm vasculature (Panza et al., 1990), and blunted acetylcholine-induced release of endothelium-dependent relaxing factor (Linder et al., 1990) in hypertensive patients.

The mechanisms by which hypertension causes endothelial dysfunction were evaluated in a myriad of studies, mostly using preclinical models. Sustained elevation of systemic pressure in the microvasculature leads to premature aging and increased turnover of endothelial cells, impairing the ability of endothelium to release endothelium-derived relaxing factors, resulting in vasoconstriction (Konukoglu and Uzun, 2017). Mechanical stress evoked by high intraluminal pressure on the vascular wall activates NADPH oxidase (NOX), which is the major ROS-producing enzyme. Excessive ROS production triggers oxidative stress that leads to endothelial dysfunction (Carmine et al., 2009). Oxidative stress incites a destructive cascade identified at the arterial wall, followed by chronic inflammation (Castellon and Bogdanova, 2016). Chronic inflammation results in changes in the arterial wall, such as geometric vascular remodeling, increase in intima-media thickness and functional remodeling (Castellon and Bogdanova, 2016). With time, these developments lead to loss of homeostatic properties, a key role in protection against endothelial dysfunction (Castellon and Bogdanova, 2016).

Endothelial dysfunction is suggested to be involved in the progression of COVID-19 because of the atypical manifestations among patients such as cardiac injury (Wang et al., 2020a) and hypercoagulability as measured by an increase in D-dimer and von Willebrand factor (VWF) levels (Spiezia et al., 2020). A recent study found that 72% of deaths due to COVID-19 had evidence of hypercoagulability (Tang, 2020). Another study found a significantly elevated level of VWF in patients with COVID-19 that lend credence to the SARS-CoV-2-induced endothelial dysfunction hypothesis (Panigada et al., 2020). Common inflammatory markers seen in endothelial dysfunction (Kaur et al., 2018) including C-reactive protein (CRP), IL-6, interferon gamma-induced protein-10 (IP-10), monocyte chemoattractant protein-1 (MCP-1), macrophage inflammatory protein-1 alpha (M1P1A), and TNF-α were also elevated in patients with COVID-19 (Zhou et al., 2020).

The inflammatory cytokines such as TNF-α, IL-1β, and IL-6 induce the synthesis of acute phase proteins by the liver, including fibrinogen (Colantuoni et al., 2020), thereby producing a pro-coagulant state (Lazzerini et al., 2020). Furthermore, the influx of activated neutrophils is prone to aggregate and form neutrophils extracellular traps (NETs) at high cellular densities (Schauer et al., 2014). NETs aggregate and occlude blood vessels (Jiménez-Alcázar et al., 2017). Subsequently, the pro-adhesive and pro-thrombotic endothelium stimulates further adhesion of leukocytes and platelets to the endothelium, causing vascular micro-thrombosis, capillary plugging, and impaired capillary flow (DiGiandomenico et al., 2014). This explains the high rate of deep venous thrombosis complicated by pulmonary embolism, myocardial infarction, stroke, and critical limb ischemia in patients with COVID-19 (Bikdeli et al., 2020).

Cytokine storm, an expression of exaggerated host immune system response, was reported in patients with COVID-19. The cytokine storm is characterized by very high levels of erythrocyte sedimentation rate (ESR), CRP, TNF-α, IL-1β, IL-1RA, IL-2 (Li et al., 2020), IL-6, IL-7, IL-8, IL-9, IL-10, granulocyte-colony stimulating factor (GCSF), IP-10, MCP-1, and MIP1 (Zhang et al., 2020b). The cytokine storm has also been described in other coronavirus pneumonia, such as SARS-CoV-1 and MERS-CoV, leading to ARDS (Dashti-Khavidaki and Khalili, 2020; Zhang et al., 2020d), and death (Channappanavar and Perlman, 2017; Chousterman et al., 2017). The cytokine levels in severe COVID-19 infection were reported to be higher than SARS and MERS (Conti et al., 2020; Huang et al., 2020). In particular, IL-6 level was the highest in severely ill COVID-19 patients (Chen et al., 2020; Ruan et al., 2020; Wu et al., 2020; Zhang et al., 2020d; Zhou et al., 2020). Elevated cytokines initiate the influx of various immune cells such as macrophages, neutrophils, and T cells from the circulation to the site of infection, causing destabilization of endothelial cell–cell interactions, capillary damage, diffuse alveolar damage, multiorgan failure, and ultimately death (Ragab et al., 2020). Acute lung injury is a consequence of the cytokine storm that can progress into its severe form, ARDS (Shimizu, 2019).

## Sars-CoV-2 and Ras Dysregulation in Hypertensive Patients

Dysregulation of RAS, which consists of angiotensinogen, angiotensin-generating enzymes, and angiotensin, as well as their receptors (Leung, 2010) is one of the clinical implications of SARS-CoV-2 infection. The components of RAS have been reported to be present locally in many organs such as the heart, lungs, and liver, which function through autocrine and paracrine mechanisms, independent of the circulating RAS (Paul et al., 2006). The organ-based or local RAS plays a specific role in injury or repair response, inflammation, and fibrosis pathways.

The SARS-CoV-2 entry into host cells via ACE2 downregulates the membrane-bound ACE2, hence causing concurrent loss of catalytic activity of ACE2 in the RAS (Verdecchia et al., 2020). Reduction in ACE2 level causes Ang II upregulation and overactivity of the conventional ACE/Ang II/AT_1_R axis. Consequently, Ang 1-7 decreases and this lessens the protective effects of the non-conventional ACE2/Ang 1-7/Mas axis. Since Ang II has pro-oxidative and pro-inflammatory actions, excessive Ang II promotes endothelial dysfunction and cytokine storm that subsequently led to the pulmonary, inflammatory, and hematological complications of COVID-19 (Costa et al., 2020; Vieira et al., 2021).

Ang II via AT_1_R stimulates ROS production in the lung’s endothelium by increasing the expression and catalytic activity of the NOX family proteins (Brown and Griendling, 2015). ROS are also produced by immune cells in response to Ang II stimulation. Excessive ROS results in the uncoupling of eNOS to a state where the enzyme no longer produces NO but instead produces superoxide. Reduced NO bioavailability leads to endothelial dysfunction as manifested by the pro-inflammatory, pro-adhesive, and pro-thrombotic endothelium (Brown and Griendling, 2015; Sundar et al., 2019). Additionally, Ang II also triggers endothelial inflammation. Ang II activates the nuclear factor κB (NF-κB) cascade, induces cellular adhesion molecules expression, and enhances leukocyte-endothelial interaction (Marchesi et al., 2008; Scalia et al., 2011). Vascular permeability increases and a large amount of fluid and blood cells enter the alveoli, leading to dyspnea and respiratory failure (Channappanavar and Perlman, 2017).

The binding of SARS-CoV-2 to alveolar epithelial cells leads to activation of the innate and adaptive immune systems, followed by the release of several cytokines, including IL-6. Previous studies showed that some viral products promote the DNA-binding activity of NF-κB and nuclear factor IL-6 (NF-IL-6) that upregulate IL-6 mRNA transcription (Tisoncik et al., 2012; Tanaka et al., 2014). Inflammation and immune response start at the initial stage of COVID-19, but as the infection progresses, these mechanisms further intensify. The exaggerated inflammatory response and intrusion of hepatocytes by SARS-CoV-2 might cause liver injury (Zhang et al., 2020a). IL-6 stimulates the release of several acute phase proteins, CRP and fibrinogen and reduces the production of fibronectin, albumin, and transferrin by the liver (Tanaka et al., 2014). Increased D-dimer levels and prothrombin time and reduced activated partial thromboplastin time are some laboratory findings related to endothelial dysfunction and liver injury due to the exaggerated inflammation. These subsequently create a hypercoagulable state with an increased risk of thrombotic and thromboembolic events (Huang et al., 2020; Tang, 2020; Wu et al., 2020).

The final stage of COVID-19 disease progression is marked by a systemic hyper-inflammatory state known as Cytokine Storm Syndrome (Mehta et al., 2020). Uncontrolled cytokines release causes the influx of various immune cells such as macrophages, neutrophils, and T cells from the circulation to the site of infection, causing destabilization of endothelial cell-cell interactions, capillary damage, diffuse alveolar damage, multiorgan failure, and eventually death (Ragab et al., 2020).

The susceptibility of hypertensive patients to severe COVID-19 outcome and the fact that SARS-CoV-2 uses ACE2 to enter the cells raised a question regarding the possibility of RAS components modification by antihypertensive agents, with angiotensin-converting enzyme inhibitors (ACEi) and angiotensin receptor blockers (ARBs) being in the center of interest (Wang et al., 2020c). ACEi effectively inhibits ACE activity without affecting Ang II binding to AT_1_R. Upon ACE inhibition, Ang I is unable to be converted into Ang II efficiently, thus less Ang II is available to bind to AT_1_R. Whereas ARBs suppress the binding of Ang II to AT_1_R, thus preventing the harmful effects of Ang II (Leclézio et al., 2020).

Animal study showed that reduced plasma Ang II level following ACEi therapy is a stimulus for the upregulation of ACE2 mRNA expression in rats (Ferrario et al., 2005). Even though ARBs have a different mechanism of action than ACEi, ARBs also increase ACE2 expression. There was a five-fold increase in ACE2 level following treatment with lisinopril, and a three-fold increase in ACE2 level with losartan treatment (Ferrario et al., 2005). This raised the argument whether it is safe to use ACEi/ARBs to manage hypertension in patients with COVID-19, considering that higher expression of ACE2 leads to increased availability of the binding sites for the SARS-CoV-2 entry into cells (Rico-Mesa et al., 2020). However, it needs to be highlighted that the study was conducted in rats and only cardiac ACE2 mRNA level was measured. More recent human studies showed no significant difference of ACE2 expression in the kidneys and lungs of patients receiving ACEi and ARBs treatment (Jiang et al., 2020; Lee et al., 2020).

A prospective, randomized, open-label clinical trial was conducted to evaluate the effect of continuation versus discontinuation of RAS inhibitors on hospitalized COVID-19 patients. No effect on severity of COVID-19 infection was observed between the groups (Cohen et al., 2021). Subsequently, a cohort study involving two million hypertensive patients found that ACEi and ARBs were associated with a lower risk of COVID-19 hospitalization compared with calcium channel blockers (CCBs). Moreover, patients taking ACEi and ARBs had a lower risk of intubation and death compared to those taking CCBs (Laura et al., 2021).

With all these uncertainties and multiple theories regarding the role of RAS inhibition in COVID-19 infection, withdrawal of ACEi/ARBs would lead to more harm than benefit in critically ill patients with multiple comorbidities (Kunal et al., 2020). As a result, various cardiovascular societies such as European Society of Cardiology have come forward and issued statements regarding continuation of ACEi and ARBs in these patients (de Simone, 2020). The International Society of Hypertension (ISH) further endorsed the statements that there was no substantial evidence to avoid ACEi or ARBs for managing hypertension in COVID-19 patients (ISH, 2020).

## Conclusion and Future Directions

**FIGURE 3:**
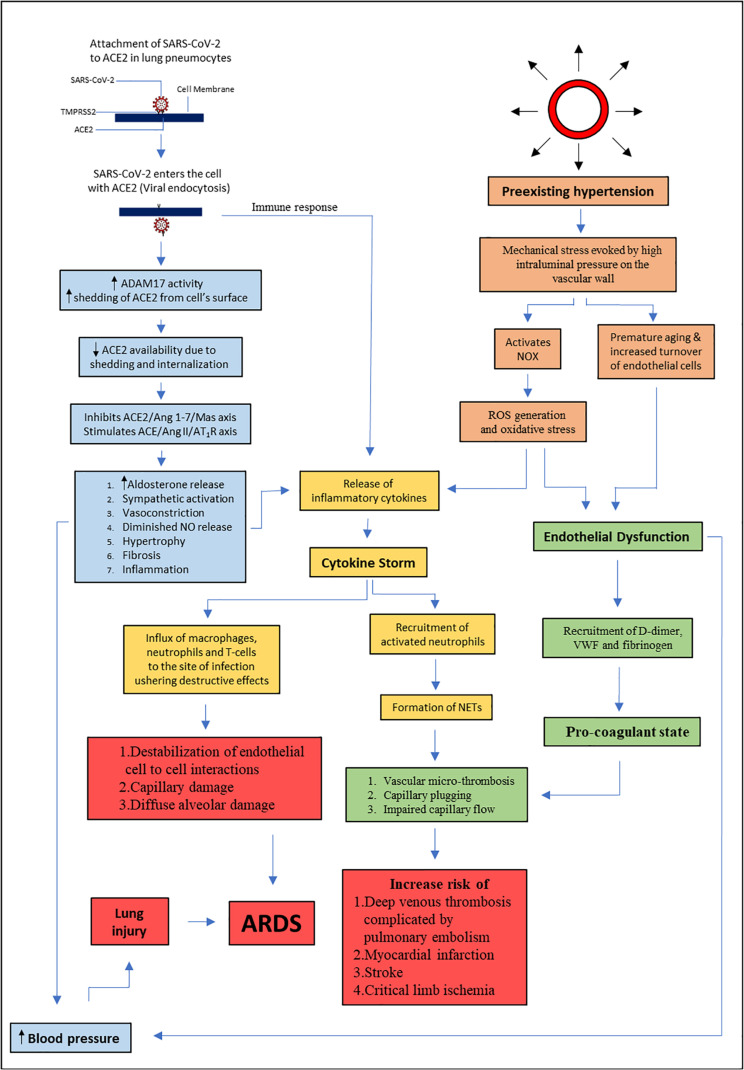
Summary of the link between hypertension and COVID-19. This diagram visualizes the relationship between SARS-CoV-2 infection, RAS dysregulation, and endothelial dysfunction. ACE : angiotensin converting enzyme, ACE 2: angiotensin converting enzyme 2, Ang 1-7: angiotensin 1-7, Ang II: angiotensin 2, AT_1_R: angiotensin II type 1 receptor, NO: nitric oxide, ARDS: acute respiratory distress syndrome, SARS-CoV-2: severe acute respiratory distress coronavirus 2, TMPRSS2: transmembrane protease serine 2, ADAM17: ADAM metallopeptidase domain 17, NOX: nicotinamide adenine dinucleotide phosphate oxidase, ROS: reactive oxygen species, VWF: von Willebrand factor, NETs: neutrophil extracellular traps.

Hypertensive patients are more vulnerable to develop serious complications of COVID-19. Figure 3 summarizes the link between hypertension and COVID-19 that involves endothelial dysfunction and RAS dysregulation. SARS-Cov-2 entry to the host cell involves ACE2, which is an important enzyme in blood pressure homeostasis. Therefore, modification of RAS may affect the development and progression of COVID-19. Early hypothesis describing exacerbation of the disease following the use of antihypertensive medications such as ACEi and ARBs was not clinically proven. To date, there are no clinical data to implicate ACEi or ARBs in either improvement or worsening of COVID-19 cases, or as a risk factor for COVID-19 infection. There is also no substantial evidence to support discontinuation of ACEi or ARBs or alternate pharmacotherapy to manage hypertension in patients with COVID-19. Large studies that consider all potential sources of biasness and confounding factors are warranted in near future to affirm the link between preexisting hypertension and COVID-19 severity and to devise better pharmacological management of COVID-19 patients with hypertension.

## Author Contributions

S-AM, AU, JK, DS, AH, and AA contributed to the writing of the original draft. All authors contributed to the article and approved the submitted version.

## Conflict of Interest

The authors declare that the research was conducted in the absence of any commercial or financial relationships that could be construed as a potential conflict of interest.
